# The role of pre-emptive culling in the control of foot-and-mouth disease

**DOI:** 10.1098/rspb.2009.0427

**Published:** 2009-07-01

**Authors:** Michael J. Tildesley, Paul R. Bessell, Matt J. Keeling, Mark E. J. Woolhouse

**Affiliations:** 1 Centre for Infectious Diseases, University of Edinburgh, Ashworth Laboratories, Kings Buildings, West Mains Road, Edinburgh EH9 3JT, UK; 2 Department of Biological Sciences, University of Warwick, Gibbet Hill Road, Coventry CV4 7AL, UK

**Keywords:** foot-and-mouth, modelling, control

## Abstract

The 2001 foot-and-mouth disease epidemic was controlled by culling of infectious premises and pre-emptive culling intended to limit the spread of disease. Of the control strategies adopted, routine culling of farms that were contiguous to infected premises caused the most controversy. Here we perform a retrospective analysis of the culling of contiguous premises as performed in 2001 and a simulation study of the effects of this policy on reducing the number of farms affected by disease. Our simulation results support previous studies and show that a national policy of contiguous premises (CPs) culling leads to fewer farms losing livestock. The optimal national policy for controlling the 2001 epidemic is found to be the targeting of *all* contiguous premises, whereas for localized outbreaks in high animal density regions, more extensive fixed radius ring culling is optimal. Analysis of the 2001 data suggests that the lowest-risk CPs were generally prioritized for culling, however, even in this case, the policy is predicted to be effective. A sensitivity analysis and the development of a spatially heterogeneous policy show that the optimal culling level depends upon the basic reproductive ratio of the infection and the width of the dispersal kernel. These analyses highlight an important and probably quite general result: optimal control is highly dependent upon the distance over which the pathogen can be transmitted, the transmission rate of infection and local demography where the disease is introduced.

## 1. Introduction

The control options for infectious diseases of livestock commonly include culling of both infected animals and animals considered to be at increased risk of infection, the latter referred to as ‘pre-emptive’ culling. Within the last decade, such strategies have been deployed in Europe, North America and Asia to control diseases such as avian influenza, bovine tuberculosis, bovine spongiform encephalopathy, classic swine fever and foot-and-mouth disease (FMD) (Keeling *et al.* 2001; Klinkenberg *et al.* 2003; Donnelly *et al.* 2006; Savill *et al.* 2006, 2008). On occasion, the culling programmes can be very extensive, involving millions of animals on thousands of farms. One well-known and much discussed example of culling to control livestock disease occurred during the UK 2001 epidemic of FMD. This was an exceptionally well-recorded epidemic, providing valuable data on the spread of an infection between farms over a complex landscape. Although there have been several detailed analyses of these data (Ferguson *et al.* 2001; Keeling *et al.* 2001; Chis Ster *et al.* 2009; Deardon *et al.* in press), there remains some controversy over the true impact of the culling programmes introduced at the time (e.g. Kitching *et al.* 2007; Tildesley *et al.* 2007). The UK 2001 FMD data provide an opportunity to explore the expected impact of different culling strategies, particularly the extent of pre-emptive culling and how best to target the pre-emptive culling effort (echoing previous work asking the same questions with regard to reactive vaccination programmes: Keeling *et al.* 2003; Tildesley *et al.* 2006).

This paper has three elements. First, we present data on the culling programme implemented during the UK 2001 FMD epidemic, paying particular attention to how pre-emptive culling was targeted. Second, we use an adapted version of a stochastic spatio-temporal farm-based model (Keeling *et al.* 2001) to carry out a retrospective model-based analysis of the 2001 epidemic to estimate the impact of pre-emptive culling in practice. Finally, the model is used prospectively to examine the effect of different culling strategies on controlling FMD outbreaks in general, considering both variations in the transmissibility of disease and the regions of the UK in which it is introduced.

## 2. The 2001 FMD epidemic

During 2001, the UK experienced an epidemic of FMD that lasted seven months with disease reported on some 2026 infected premises (IPs). In addition to the 2026 IPs, 250 farms were culled as suspected FMD cases, and animals on a further 8570 premises were culled pre-emptively. These data were recorded in the disease control system (DCS) database and the reasons for the pre-emptive culls can be broken down into two main categories.

### (a) Culls of farms ‘at risk’ (5312 farms)

Farms at elevated risk of harbouring disease were identified on a case-by-case basis and were culled accordingly. Such farms were officially designated as either traditional ‘dangerous contacts’ (DCs) or ‘contiguous premises’ (CPs). DCs were defined as ‘premises where animals have been in direct contact with infected animals or have, in any way, become exposed to infection’ and CPs as ‘a category of dangerous contacts where animals may have been exposed to infection on neighbouring infected premises’ (Anderson 2002). These two kinds of pre-emptive cull were imperfectly distinguished in practice (in principle some farms could have been culled under either heading, with such farms sometimes being recorded as a DC, sometimes as a CP and sometimes as ‘other’). CP culling was officially introduced on 27 March 2001, and partly relaxed from 26 April 2001 by allowing the exemption of some cattle and rare breeds from culling. Local discretion in CP culling was also permitted (Honhold *et al.* 2004)—veterinary inspectors were given the power to cull only parts of a holding if it was felt the entire holding had not been exposed (National Audit Office 2002). In practice, CP culling was never fully implemented and not all contiguous farms had their livestock culled (National Audit Office 2002).

### (b) Three kilometre cull and local culls (3260 farms)

A cull of 700 000 sheep on 2000 farms in north Cumbria and south west Scotland was approved on 15 March 2001 and formally implemented from 22 March 2001 (National Audit Office 2002). These holdings lay within 3 km of an IP and were thought to be at elevated risk of already being infected from the initial seed at Longtown market (National Audit Office 2002; Thrusfield *et al.* 2005). The 3 km cull ended in mid May, although it was never implemented fully in Cumbria (National Audit Office 2002). Local culling principally occurred in northwest Wales where all farms that had purchased sheep from the Welshpool market (one of the early nodes from which infection spread) during the ‘at-risk’ period had livestock culled.

The rationale for the ‘at risk’ and 3 km/local culls was to target premises that were harbouring undetected infection. Culling uninfected farms was not an explicit aim of any of the forgoing control strategies, however, it should be noted that removing farms that are not infected can help to control the epidemic by reducing the local density of susceptible farms, which reduces the local reproductive ratio. It was anticipated that of those farms that were infected, many farms would have been ‘pre-clinical’—the animals were too early in the course of infection to display clinical signs. These farms were the main target of ‘at-risk’ culling, requiring the identification of farms with elevated risk of having been exposed to infection. Local and 3 km culls, as well as removing pre-clinical farms, removed holdings on which clinical signs of disease had been missed or on which animals had become infected and recovered without the farm being reported. In addition to the culls for disease control purposes mentioned here, 1.8 million sheep, 166 000 cattle and 306 000 pigs were culled for welfare reasons (Anderson 2002). Welfare culling was not targeted at farms at elevated risk of infection and constituted a relatively small fraction of the farms culled in areas directly affected by FMD; welfare culls are not considered further here.

## 3. Data analysis

Using the data from the DCS, we evaluated the numbers of animals culled by species in each of the two cull categories defined earlier. European Union (EU) policy in 2001 stated that in the event of an outbreak of FMD, a 3 km protection zone and a 10 km surveillance zone (SZ) should be set up around all IPs. When analysing the 2001 epidemic, we also needed to take into account the underlying demographic data (taken from the June 2000 agricultural census of England, Scotland and Wales) and in line with EU policy, we considered all farms within the SZs during the 2001 epidemic.

During the epidemic, disease control centres (DCCs) were responsible for control of the spread of disease within a local region. In 2001, there were 18 such DCCs, although many of these handled very few IPs. The DCCs managing the largest proportion of IPs in 2001 were Carlisle (891 IPs), Newcastle (191), Ayr (177), Exeter (172) and Leeds (139), while the remaining 13 DCCs all managed fewer than 100 IPs. We ignore the 250 suspected FMD cases, as these did not trigger any pre-emptive culling.

For the purposes of this analysis, the 2001 epidemic can be divided into three ‘phases’. Phase 1 was defined as the period before 26 March (prior to implementation of CP culling), phase 2 was the period of full CP culling up to 29 April and phase 3, the period of reduced CP culling that followed until the end of the epidemic on 30 September 2001. We then looked for differences in the implementation of all pre-emptive culling dependent upon DCC and epidemic phase. We also analysed the ratio of cattle to sheep culled on IPs and non-IPs relative to the background population. The ratios on all culled farms were compared with the ratios for IP culls and non-IP culls and the background demography calculated as the total of all holdings within the 10 km SZ of all IPs.

**Figure 1.**
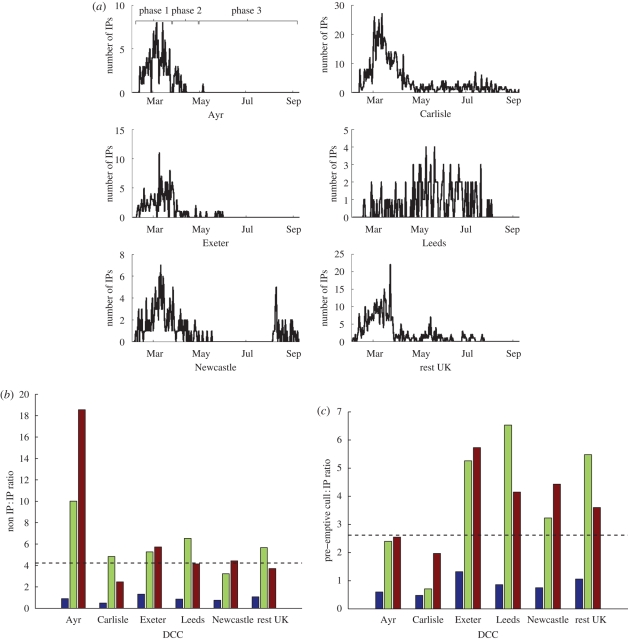
(*a*) Temporal pattern of IPs in the five DCCs with the greatest number of IPs (and in the rest of the UK), (*b*) non-IP to IP ratio and (*c*) pre-emptive cull to IP ratio, for these DCCs during the three phases of the epidemic. (*b*,*c*) The dashed lines show the non-IP to IP ratio and the pre-emptive cull to IP ratio respectively for the whole country, averaged over the entire epidemic. Blue bar, phase 1; green bar, phase 2; brown bar, phase 3.

Of all farms culled for disease control purposes in 2001, only 18.6 per cent were IPs. The regional and temporal variation in cases and culls is illustrated in figure 1. Of the five DCCs used in these analyses, Exeter, Ayr and Carlisle followed a similar epidemic curve to the remainder of the country (figure 1*a*); Newcastle was not a self-contained epidemic in the same way as the disease was repeatedly reintroduced from outside, while some farms in the Leeds DCC are thought to have harboured latent infection until the end of April when the epidemic took off following the release of cattle to pasture.

Typically, there was less than 1 ‘at risk’ cull per IP in all DCCs (except Exeter) in phase 1 (figure 1*b*). During phase 2, pre-emptive culls rose to around 3–7 per IP in the DCCs that did not implement 3 km culling (mainly through the introduction of CP culling) and rose to over 4 in those DCCs that did (Ayr and Carlisle). During phase 3, 3 km culling was largely brought to an end in Carlisle with other pre-emptive culls continuing at a rate of 2 per IP, lower than anywhere else in the UK. From this, we see that there were marked differences in the intensity of ‘at-risk’ and 3 km/local culling both through time (phase) and between regions (DCCs). For the ‘at-risk’ (DC + CP) culling of interest here, the most striking observation is that the culling intensity was consistently lowest for the Carlisle DCC (figure 1*c*), followed by the Ayr DCC (but there offset by the extensive 3 km culls).

**Figure 2.**
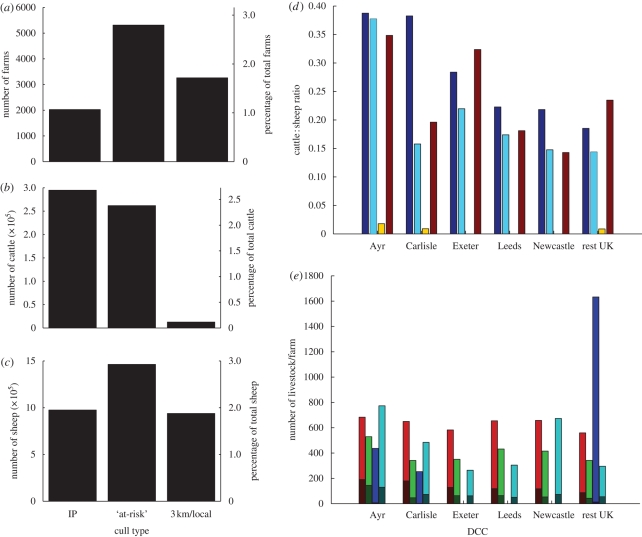
(*a*) Number of farms losing livestock, (*b*) number of cattle culled and (*c*) number of sheep culled for the various cull categories used during the 2001 FMD epidemic. ‘At-risk’ culls include all farms culled in the DCS categories: traditional dangerous contacts (DCs), CPs and others. The right-hand axis in each figure shows the percentage of the total population in the UK of farms, cattle and sheep respectively culled in each category. (*d*) Cattle-to-sheep ratio on IPs, ‘at-risk’ culls and 3 km/local culls for individual DCCs. The cattle : sheep ratio for all farms within the 10 km surveillance zone (SZ) of all IPs in each DCC is also shown (navy blue bar, IPs; sky blue bar, ‘at-risk’; yellow bar, 3 km + local; brown bar, census 10 km SZ). (*e*) The number of cattle and the number of sheep in each DCC on IPs, ‘at-risk’ culls, 3 km/local culls and in the 10 km SZ of all IPs (brown bar, cattle IPs; red bar, sheep IPs; green bar, cattle ‘at-risk’; light green bar, sheep ‘at-risk’; indigo bar, cattle 3 km + local; navy blue bar, sheep 3 km + local; dark green bar, cattle census 10 km SZ; sky blue bar, sheep census 10 km SZ).

A variety of analyses have previously shown that farms with many animals are at higher risk (both in terms of transmission and susceptibility) compared with smaller farms, and that cattle farms are at higher risk than sheep farms (Ferguson *et al.* 2001; Keeling *et al.* 2001; Deardon *et al.* in press); it is therefore important to examine the number of animals as well as the cattle : sheep ratio associated with any cull. In 2001, there were 2026 IPs, 5312 ‘at-risk’ culled farms and 3260 farms were part of the 3 km and local culling policies described earlier (figure 2*a*). Nationally, nearly 300 000 cattle were culled on IPs, corresponding to 50.7 per cent of all cattle culled during the epidemic (and just over 2.5% of the national cattle herd; figure 2*b*) while 28.0 per cent of all sheep culled were on IPs (just under 2% of the national sheep flock; figure 2*c*); 25.4 per cent of all sheep culled were removed during the 3 km cull in Cumbria and Dumfriesshire, compared with only 1.9 per cent of cattle, owing to the fact that the 3 km cull was aimed specifically at sheep farms. In total, 8.8 per cent of the national sheep flock was culled compared with 6.1 per cent of the national cattle herd. Cattle : sheep ratios were much lower on 3 km and local farms than on IPs for all DCCs implementing these strategies (figure 2*d*). In all DCCs except Ayr and Newcastle, the cattle : sheep ratio on ‘at-risk’ culls was found to be lower than on IPs and on all farms within the SZs (according to the 2000 census data). In Newcastle, the cattle : sheep ratio on ‘at-risk’ farms was only slightly higher than in the SZs and much lower than on IPs. Only in the Ayr DCC was the cattle : sheep ratio on ‘at-risk’ farms almost equal to that on IPs and greater than in the SZs.

In all DCCs, the average number of cattle culled per farm was much greater on IPs than on ‘at-risk’ culls and on farms within the SZs (figure 2*e*). ‘At-risk’ culling was targeted towards farms with fewer cattle and sheep than was found on IPs in all DCCs. This effect was particularly noticeable in Ayr and Carlisle, where the number of livestock per farm was substantially lower on ‘at-risk’ culls than in the SZs. Very few cattle were removed as part of the 3 km and local culls.

In summary, our analysis of the DCS data highlights two important features of the pre-emptive culling carried out during the 2001 epidemic. First, pre-emptive culling was typically targeted at sheep farms and/or at sheep on mixed farms. This is in contrast with the observation that IPs tended to be cattle farms or farms with cattle, supported by subsequent analyses indicating that numbers of cattle were a major risk factor (Keeling *et al.* 2001; Chis Ster *et al.* 2009; Deardon *et al.* in press; Bessell *et al.* in press). Second, the overall pre-emptive culling effort and the targeting of pre-emptive culling were highly variable both through time (in response to changing national directives) and across DCCs. The Carlisle DCC was notable both for having the lowest ‘at-risk’ culling effort (figure 1) and for targeting low-risk sheep farms rather than high-risk cattle farms (figure 2).

To analyse the culling strategies used in 2001 to investigate the effect of CP culling in various regions of the UK and devise optimal culling strategies for control of the 2001 epidemic and potential future infections of FMD in the UK, we adopted a mathematical modelling approach.

## 4. The model

For this analysis, we used an adapted version of the model developed by Keeling *et al.* (2001) during the 2001 FMD epidemic. The model takes the same form as in previous papers (Tildesley *et al.* 2008), such that the rate at which an infectious farm *i* infects a susceptible farm *j* is given by
4.1
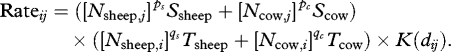



*N*_*s,i*_ is the number of livestock species *s* recorded as being on farm *i*, *S*_*s*_ and *T*_*s*_ measure the species-specific susceptibility and transmissibility, *d*_*ij*_ is the distance between farms *i* and *j* and *K* is the distance-dependent transmission kernel, estimated from contact tracing (Keeling *et al.* 2001). Here *p*_*s*_, *p*_*c*_, *q*_*s*_ and *q*_*c*_ are power-law parameters accounting for a non-linear increase in susceptibility and transmissibility as animal numbers on a farm increase. Previous work has found that this power-law model provides a closer fit to the 2001 data than one in which the powers are set to unity (Diggle 2006; Tildesley *et al.* 2008; Deardon *et al.* in press). All model parameters are estimated from the 2001 epidemic data and are determined for five distinct regions: Cumbria, Devon, the rest of England (excluding Cumbria and Devon), Wales, and Scotland, allowing us to account for regional variation in FMD epidemiology.

The UK livestock census database defines the farm location as a single point, which is usually the location of the farmhouse. Contiguous farms are, in practice, defined as farms that share a common boundary, determined on an individual case-by-case basis using local knowledge and maps where available. In our model, we determine contiguous farms by tessellating around each farmhouse point location, taking into account the known area of each farm, and therefore obtain a surrogate set of adjacent farms. As discussed elsewhere (Ferguson *et al.* 2001; Kao 2003; Tildesley *et al.* 2008), many premises in the UK are made up of multiple land fragments, with highly fragmented farms generally having a higher number of associated CPs. In the census database, some fragments have unique identifiers (defined as county-parish-holding or ‘CPH’ numbers), and our tesselation method will calculate CPs around these fragments. While this set of farms does not necessarily correspond to the true set of CPs, it will capture many of the elements of local proximity (Keeling *et al.* 2001).

In practice, not all farms that were contiguous to infected farms were culled during 2001 and, as shown earlier, culling was often targeted at sheep rather than cattle. Therefore in our model, upon introduction of CP culling, we do not necessarily cull all farms found to be contiguous to IPs. Rather, we introduce a region- and time-specific CP culling parameter that allows us to vary the proportion of CP culling that takes place. Based upon the region and time point of the epidemic, each farm estimated to be contiguous to an infected farm is allocated a probability of being removed as a CP farm—this probability is determined from the 2001 epidemic data and takes a value of between 0.7 and 0.9 dependent upon region. This allows the model to capture the regional differences in pre-emptive culling effort observed in practice (figure 2). In addition, we vary the relationship between the risk that a farm is infected and the probability that it is culled as a CP. We consider five scenarios: (i) no CP culling; (ii) random selection from possible CPs; (iii) choosing farms at lowest risk of infection (determined by equation 4.1); (iv) choosing farms at highest risk of infection; and (v) culling all CPs. In addition, we consider a related strategy, ring culling. We choose the radius of the ring from a large number of simulations to minimize the epidemic impact (see the following). We assume that a maximum of 100 farms can be ring culled per day, in line with previous work (Tildesley & Keeling 2008). For random, lowest-risk and highest-risk culling of CPs, only a fraction of the total CPs are culled, with this fraction following the observed pattern in 2001.

Other kinds of culls—DCs, 3 km and local culls—are modelled in the same way as in previous analyses (Tildesley *et al.* 2006, 2008). During the 2001 epidemic, DCs were identified for each IP on a case-by-case basis, using veterinary judgement of risk factors and known activities, such as the movement of vehicles. In our model, DCs are determined stochastically, such that the probability that farm *i* is a DC associated with IPs *j* is given by

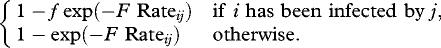

The parameter *f* controls the accuracy of DC culling—the ability to detect routes of transmission—while *F* governs the overall level of DC culling per reported case; *F* is allowed to vary through time to reflect the changing levels of DC culling that occurred during the epidemic, while *f* is another free parameter that needs to be estimated. Best-fit values for *F* and *f* are obtained from the 2001 tracing data—*F* takes values between 3.5 and 9.0, while *f* takes values between 0.84 and 0.90, dependent upon the region of the UK. We use the same spatial kernel to assign infection and for the identification of DCs, although in principle, it may be possible to estimate different kernels reflecting any biases in DC ascertainment.

In 2001, there was a target of culling all IPs within 24 h of reporting infection and associated pre-emptive culling within 48 h, but this was rarely achieved in practice. We began our analysis by using the distribution of culling delays as observed during the 2001 epidemic. However, to explore the importance of the 24/48 h target, we repeated our simulations but replaced the 2001 distributions with fixed 24 and 48 h delays as intended.

We next considered the effect of CP culling on outbreaks in various regions throughout the UK, with emphasis on Cumbria, which was the major ‘hot spot’ during the 2001 epidemic. Culling was again fixed at 2001 levels, although a 24/48 h culling policy was assumed. In the Cumbrian scenario, it was assumed that the epidemic occurred from a single infectious source with silent spread to a further 19 farms prior to the first case being reported, simulating a clustered outbreak.

For all the earlier simulations, we quantified the effectiveness of the control strategy using two indicators. First, we calculated the ‘epidemic impact’, the total number of farms that were culled, whether as IPs or as pre-emptive culls; second, we calculated the duration of the epidemic.

It is possible that optimal culling strategies are highly influenced by the epidemiological properties of the infecting FMD strain. With this in mind, a sensitivity analysis was carried out. The height (*K*_h_) and width (*K*_w_) of the dispersal kernel were allowed to vary, to investigate the effect of optimal control on future epidemics in which the total transmission rate of the virus and the distance over which the virus could be transmitted varies. Six different culling strategies were investigated: no CP culling, random CP culling, CP culling targeted towards the highest-risk farms, CP culling targeted towards the lowest-risk farms, culling all CPs, and fixed radius ring culling (with no CP culls) where the radius was optimized to minimize the epidemic impact for each strategy. In each case, IPs and DCs were culled routinely and 24/48 h delays were assumed.

Finally, building on the earlier results for individual counties, we developed spatial maps to quantify the effect of CP culling on outbreaks from a single infectious source, similar to *R*_0_ maps previously developed to estimate epidemic risk (Boender *et al.* 2007; Racloz *et al.* 2008). We seeded an outbreak in each county in the UK in turn and ran 10 000 simulations for all counties, allowing the levels of CP culling to vary across simulations. This enabled us to calculate the optimal level of CP culling that would be needed to be achieved to combat local epidemics.

## 5. Results

Using 2001 initial conditions, if CP culling had not been carried out, with other culls at 2001 levels, we found the largest epidemic impact and longest duration of all the control strategies considered (and much higher than that which actually occurred during 2001; table 1, top section). We note that even if CP culling was targeted towards the lowest-risk farms, the epidemic impact was, on average, lower by 1700 farms than with no CP culling (table 1, top section). With an efficient 24/48 h culling policy, the same result was found, but now the average epidemic impact was further reduced by around 2000 farms (table 1, middle section). For both scenarios, the optimal strategy was to target all CP farms for culling—saving around 4000 farms on average compared with no CP culling. Poor selection of CPs during 2001 was estimated to have resulted in an epidemic involving 1000–2000 more farms and lasting up to one month longer than could have been achieved (see table 1, top section; the 2001 strategy as implemented lies between ‘with random CPs’ and ‘with lowest-risk CPs’). The optimal radius for ring culling was 0.8 km, given 2001 culling delays, and 0.75 km given 24/48 h culling. In both cases, ring culling resulted in a higher epidemic impact than well targeted CP culling but, importantly, it reduced average epidemic duration by almost two months.

**Table 1.** Mean epidemic impact and mean duration of epidemic for a range of initial conditions and culling strategies. Ninety five per cent confidence intervals on epidemic impact and duration are given in brackets. The minimum value for each set of initial conditions is highlighted in bold. All ring culling results are carried out at optimal radius for each epidemic scenario. The first row in the top section gives the epidemic impact and duration of epidemic (from the date of the introduction of movement restrictions) for the actual epidemic during 2001.

initial conditions	epidemic impact	duration
2001 epidemic	10 598	220
2001, no CPs	13 145 (9499–16 188)	346 (229–529)
2001, with random CPs	10 413 (8504–11 796)	208 (138–325)
2001, with lowest risk CPs	11 405 (9105–13 186)	234 (142–345)
2001, with highest risk CPs	9287 (7965–10 743)	191 (132–301)
2001, with all CPs	**9223** (7525–11 040)	195 (127–309)
2001, with 0.8 km ring cull	11 125 (8620–13 845)	**137** (100–202)
2001, 24/48 h culling, without CPs	11 894 (8758–14 590)	314 (148–341)
2001, 24/48 h, with random CPs	8288 (6331–10 416)	195 (100–314)
2001, 24/48 h, with lowest risk CPs	9495 (7219–11 664)	210 (121–329)
2001, 24/48 h, with highest risk CPs	7565 (5901–9654)	178 (97–289)
2001, 24/48 h, with all CPs	**7468** (5815–9211)	181 (102–296)
2001, 24/48 h, with 0.75 km ring cull	10 848 (7873–12 677)	**119** (89–189)
Cumbria, 24/48 h, without CPs	9207 (7154–14 402)	313 (218–467)
Cumbria, 24/48 h, with random risk CPs	6018 (4611–7380)	187 (104–307)
Cumbria, 24/48 h, with lowest risk CPs	7068 (5018–8940)	199 (118–321)
Cumbria, 24/48 h, with highest risk CPs	5561 (4218–6975)	167 (92–284)
Cumbria, 24/48 h, with all CPs	5514 (4058–7013)	172 (98–291)
Cumbria, 24/48 h, with 2.0 km ring cull	**4801** (3021–6383)	**112** (72–168)

If an epidemic was seeded in Cumbria, we found that (given 24/48 h culling), while well-targeted CP culling was more efficient than a policy of not culling CPs (with other control culls included), optimal radius (2 km) ring culling minimized epidemic impact in this case (table 1, bottom section), as well as minimizing epidemic duration. Cumbria was a major hot spot of infection in 2001 and, in the event of a localized epidemic, an intensive ring-culling policy could prevent infection from spreading to other counties. An intensive (optimally sized) ring-culling policy therefore had a beneficial effect within Cumbria as even full CP culling cannot generate a sufficiently high pre-emptive cull : IP ratio. Elsewhere however, ring culling is not as effective as a CP culling policy is found to naturally target more farms around high-risk IPs compared to ring culling.

The results for epidemics seeded in other counties are summarized in table 2 (comparing results for no CP culling with random CP culling). In high livestock density regions such as Cumbria, Devon, Clwyd and Dumfriesshire, CP culling reduced mean epidemic impact by reducing local densities of susceptible farms. Should an outbreak occur in low livestock density regions such as Surrey or Norfolk, CP culling would increase epidemic impact. In such circumstances, culling of traditional DC farms and IPs alone would be sufficient to contain such an outbreak. However, the disadvantages of CP culling in these regions were found to be minimal—the epidemic impact was, on average, increased by only one or two farms in each case. The effect of CP culling is found to be region-dependent—similar epidemic impacts are found in the absence of CP culling in Gwent and Fife. However, CP culling increases the epidemic impact in Gwent but reduces it in Fife; this highlights that local geography plays a significant role in the probable spread of disease and optimal control policies.

**Table 2.** Mean epidemic impact with and without CP culling for epidemics seeded in various counties in the UK. Ninety five per cent confidence intervals on epidemic impact are given in brackets. The epidemic impact of the optimum strategy for each county is given in bold.

region	epidemic impact, no CPs	epidemic impact, with CPs
Cumbria	9207 (7154–14 402)	**6018** (4611–7380)
Devon	1029 (544–5909)	**469** (289–765)
Surrey	**30** (23–35)	32 (25–38)
Norfolk	**28** (23–32)	29 (23–34)
Clwyd	889 (528–5689)	**351** (106–633)
Gwent	**123** (33–298)	139 (45–322)
Dumfriessshire	7522 (4677–12 917)	**4316** (3288–5842)
Fife	107 (27–223)	**74** (24–167)

**Figure 3.**
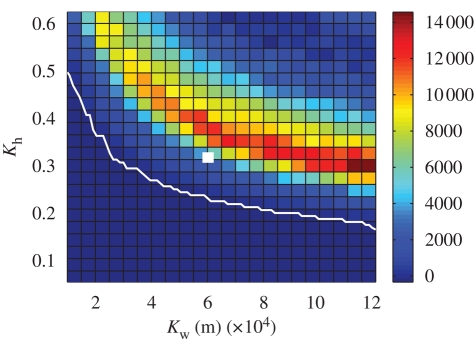
Graph showing the difference in epidemic impact between a strategy of IP and DC culling alone and a strategy of culling all CPs. The colour scale shows the number of farms ‘saved’ when all CPs are culled. The white line indicates where the two strategies result in the same overall average epidemic impact. The white box shows the point at which *K*_h_ and *K*_w_ take the values used for the 2001 epidemic.

The results of the sensitivity analysis considering a range of values of *K*_h_ and *K*_w_, seeding from 2001 initial conditions and with a 24/48 h culling policy, are shown in figure 3, evaluated in terms of epidemic impact. For low values of *K*_h_, the optimal strategy was to cull IPs and DCs alone, with no CPs and ring culls. However, when *K*_h_ reached a certain critical value, the optimal strategy switched to that of targeting all CPs. This result is unsurprising—when *K*_h_ is low, the value of *R*_0_ for the epidemic is low, and hence the epidemic impact will be restricted to a handful of farms. Any CP or ring culling will merely add to the epidemic impact without having a substantive effect on epidemic control, and hence it will be optimal to cull IPs and DCs alone. However, for epidemics with a high *K*_h_ (and hence a high value of *R*_0_), CP culling plays a crucial role in disease control and well-targeted CP culling can help to reduce epidemic size (figure 3). The colour scale shows the differences in epidemic impact for particular values of *K*_h_ and *K*_w_ with a policy of IP and DC culling alone compared with a policy of culling all CPs. For low values of *K*_h_, IP and DC culling alone was found to be optimal; however, for higher values of *K*_h_, the number of farms saved when all CP farms were targeted peaked at around 14 000 farms compared with a strategy of IP and DC culling alone. For moderate values of *K*_h_, the number of farms saved was found to increase as *K*_w_ increased. However, as *K*_h_ increased, the opposite behaviour is found—the number of farms saved by targeting all CPs as opposed to IP and DC culling alone decreased as *K*_w_ increases. When *K*_h_ and *K*_w_ are high, epidemics are highly disseminated and while CP culling does aid in control, the overall effect is reduced by the high transmission rate and the large distances over which infection can be transmitted.

**Figure 4.**
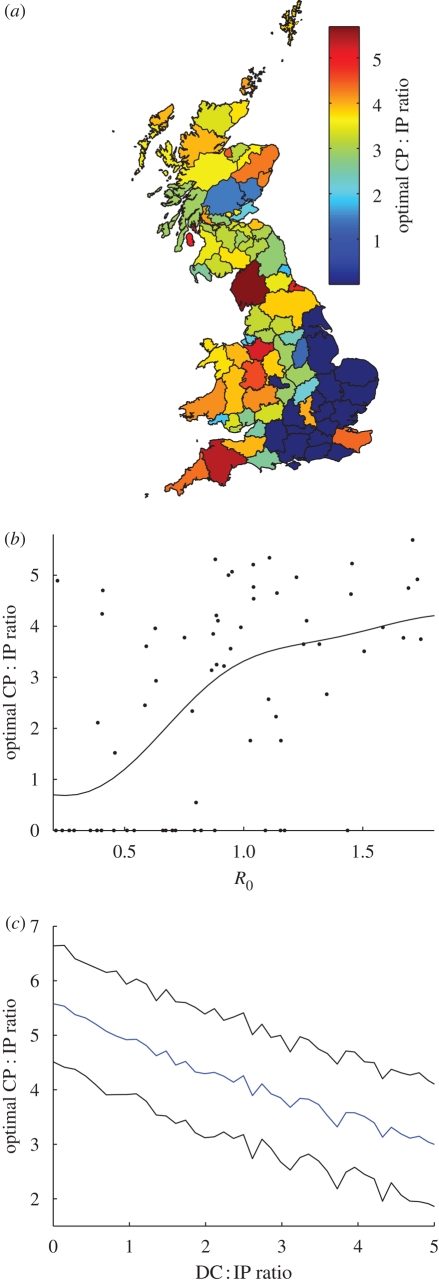
(*a*) Graph showing the effect of CP culling for epidemics seeded in each county of the UK. The colour scale shows the optimal CP : IP ratio that must be achieved to minimize the epidemic impact. (*b*) Mean optimal CP : IP ratio (black line) for epidemics seeded in each county against the mean *R*_0_ values averaged across farms in each county. The black dots show the raw data for each county. (*c*) Optimal CP : IP ratio in Cumbria as the DC : IP ratio varies (blue line). Ninety five per cent confidence intervals on the mean are also shown (black lines).

Finally, the simulations of epidemics seeded in different counties showed that, for most of the country, some level of CP culling was necessary to minimize epidemic impact, with the highest levels necessary in Cumbria, Devon, Aberdeenshire, parts of Wales and the Midlands (figure 4*a*). In the South East and East Anglia it was optimal to not employ CP culling, although the effect of CP culling on overall epidemic impact in these regions was found to be minimal. The optimal CP : IP ratio in a county was found to have a strong correlation to the mean *R*_0_ of farms in that county (figure 4*b*)—as the mean value of *R*_0_ in a county increased, the optimal CP : IP ratio to minimize epidemic impact was found to increase (cf. Matthews *et al.* 2003). Finally, we consider for Cumbria how the two forms of pre-emptive culling (CP and DC) trade-off against each other (figure 4*c*). Should DC culling not be carried out, an average of over five CPs must be culled per IP to minimize the epidemic impact. As the DC : IP ratio is increased, the optimal CP : IP ratio is found to decrease. However, even for DC : IP ratios of five (which corresponds to a very high level of success compared with that achieved during 2001) we find that it is still optimal to carry out some CP culling in Cumbria—a CP : IP ratio of around three is found to be optimal.

## 6. Discussion

Detailed analysis of the pre-emptive culls carried out during the 2001 UK FMD epidemic indicates that the culling effort was disproportionately targeted at small sheep farms and/or sheep on mixed farms, despite evidence that these were at relatively low risk after the initial dissemination of disease in February (Bessell *et al.* in press). This bias was most apparent in Cumbria, the worst affected area of the UK. This result is consistent with, and helps to explain, recent work suggesting that the proportion of pre-emptively culled farms on which infection was present was as low as a few per cent (Chis Ster *et al.* 2009; Deardon *et al.* in press). The possibility that pre-emptive culling was targeted at low rather than high-risk farms was not considered in earlier analyses of the epidemic and its control (Ferguson *et al.* 2001; Keeling *et al.* 2001), and it is important to understand its implications, particularly with respect to the controversial policy of culling farms contiguous to IPs.

Our simulation results indicate that a policy of culling *all* CPs is most effective (in terms of reducing the total number of farms culled during the epidemic). This policy performs marginally better than one in which the highest-risk farms are targeted, with the total number of CP culls set to 2001 levels. However, given the initial conditions and transmission parameters appropriate to the UK 2001 epidemic, even poorly targeted contiguous culling is beneficial. This result is in line with earlier work (Chis Ster *et al.* 2009; Deardon *et al.* in press), and we suggest that it reflects the additional benefits of reducing the local density of susceptible farms, as well as removing a small number of farms harbouring pre-clinical or sub-clinical infections. Previous work has shown that a reduction in overall density of a susceptible population does aid in disease control (Haydon *et al.* 2004; Gilligan *et al.* 2007).

If contiguous culling is implemented, there are further benefits to doing so as part of a 24/48 h strategy (i.e. prompt culling of both IPs and ‘at-risk’ farms). However, the benefits of contiguous culling are dependent both on the transmission parameters for the infection and the demography of the livestock population. Specifically, CP culling (or any other form of pre-emptive culling) is not beneficial when intrinsic transmission rates are low and/or the density of susceptible farms is low (figure 4; see also Matthews *et al.* 2003). For low transmission rates, however, the small increase in epidemic impact when CP culling is carried out highlights an important issue—the potential risk of under culling massively outweighs the risk of over culling (see Matthews *et al.* 2003). An important practical consequence of this is that for a localized outbreak, whether or not pre-emptive culling should be implemented depends on where the outbreak occurs. Maps such as that shown in figure 4*a* could be used in future to identify optimal culling policies in the event of future outbreaks of FMD.

We also considered an alternative pre-emptive culling strategy, ring culling. In some circumstances, ring culling can further reduce the total number of farms lost, although using 2001 parameters, this only occurs in Cumbria. However, if the objective of the control policy is not simply to minimize the number of farms lost but also to reduce the duration of the epidemic, then limited ring culling may be optimal. The task for policy makers is to balance the trade-off between a shorter epidemic (by as much as several months) and more farms culled (by hundreds or thousands). This is a challenging issue and we do not attempt to resolve it here.

Together, these results have implications for our understanding of the impact of the control measures implemented during the 2001 UK FMD epidemic, and more general considerations for the control of FMD in the UK and elsewhere, and for other livestock diseases where pre-emptive culling is a possible means of controlling epidemics.

Our results confirm that the introduction of contiguous culling in the UK in 2001 is expected to have reduced significantly the total number of farms lost. However, the tendency to target pre-emptive culling at low risk farms, and sheep rather than cattle, is likely to have led to the loss of 1000–2000 extra farms and to have extended the duration of the epidemic by up to a month. This problem was particularly acute in Cumbria where most of the detrimental effects were felt. Conversely, had contiguous culling in 2001 been properly targeted, prompt, and comprehensive, then there would have been substantial further reductions in the numbers of farms lost and the duration of the epidemic.

More generally, the results presented in this paper highlight an important consideration for contingency plans to combat future FMD outbreaks—initial conditions play a crucial role in determining the control strategies to be implemented. Localized outbreaks in regions of high livestock densities in general require more intense culling to minimize the number of farms affected, while in low density regions, it is probable that a large-scale outbreak will not occur, and thus, intense culling would merely add to the epidemic impact without reducing the risk of spread. Models such as the one presented in this paper can be used to develop region-specific control policies for outbreaks and thus minimize the risk of a large-scale epidemic occurring in the future.

We note that vaccination would be considered to combat outbreaks of FMD in the future, and previous work has shown that a carefully targeted vaccination strategy, combined with culling of IPs and traditional DCs, would minimize the overall epidemic impact (Tildesley *et al.* 2006). Should vaccination be carried out, CP culling tends to increase the number of farms culled as it is probable that farms targeted for CP culling would also be targeted for vaccination and hence at very low risk of subsequently becoming infected.

To conclude, we suggest that the results of this and earlier analyses based on the 2001 UK FMD epidemic (e.g. Keeling *et al.* 2003; Tildesley *et al.* 2006) illustrate some general principles of infectious disease control in heterogeneous populations. Most importantly, they indicate that reactive control policies (whether based on culling, vaccination or, in different contexts, other control options such as prophylaxis or quarantine) must pay close attention to the targeting of interventions according to the risk of current and/or future infection. Even given similar levels of effort, improved targeting can significantly reduce the scale of an epidemic and we suggest that much more attention is directed at identifying ways in which control efforts can be targeted for maximum effect.
